# Developing a Minimum Data Set of the Information Management System for Orthopedic Injuries in Iran

**DOI:** 10.5812/ircmj.17020

**Published:** 2014-07-05

**Authors:** Maryam Ahmadi, Ali Mohammadi, Ramin Chraghbaigi, Taimur Fathi, Mahdieh Shojaee Baghini

**Affiliations:** 1 Department of Health Information Management, School of Management and Medical Information Sciences, Iran University of Medical Sciences, Tehran, IR Iran; 2 Health Management and Economics Research Center, School of Health Management and Information Sciences, Iran University of Medical Sciences, Tehran, IR Iran; 3 Medical Documents Center, Social Security Organization, Kermanshah, IR Iran; 4 Department of Orthopedic, Kermanshah University of Medical Sciences, Kermanshah, IR Iran; 5 Medical Informatics Research Center, Institute of Futures Studies in Health, Kerman University of Medical Sciences, Kerman, IR Iran; 6 Department of Health Information Management, School of Paramedical, Shahid Beheshti University of Medical Sciences, Tehran, IR Iran

**Keywords:** Orthopedic, Injuries, Hospitals, Forensic Medicine, Emergency

## Abstract

**Background:**

Orthopedic injuries are the most common types of injuries. To identify the main causes of injuries, collecting data in a standard manner at the national level are needed, which justifies necessity of making a minimum data set (MDS).

**Objectives:**

The aim of this study was to develop an MDS of the information management system for orthopedic injuries in Iran.

**Materials and Methods:**

This descriptive cross-sectional study was performed in 2013. Data were collected from hospitals affiliated with Tehran University of Medical Sciences that had orthopedic department, medical documents centers, legal medicine centers, emergency centers, internet access, and library. Investigated documents were orthopedic injury records in 2012, documents that retrieved from the internet, and printed materials. Records with Random sampling by S22-S99 categories from ICD-10 were selected and the related internet-sourced data were evaluated entirely. Data were collected using a checklist. In order to make a consensus about the data elements, the decision Delphi technique was applied by a questionnaire. The content validity and reliability of the questionnaire were assessed by expert’s opinions and test-retest method, respectively.

**Results:**

An MDS of orthopedic injuries were assigned to two categories: administrative category with six classes including 142 data elements, and clinical category with 17 classes including 250 data elements.

**Conclusions:**

This study showed that some of the essential data elements included in other country’s MDS or required for organizations and healthcare providers were not included. Therefore, a complete list of an MDS elements was created. Existence of comprehensive data concerning the causes and mechanisms of injuries informs public health policy-makers about injuries occurrence and enables them to take rationale measures to deal with these problems.

## 1. Background

Injuries and their consequences are among the most important issues of modern life and the primary cause of death in those younger than 45 years of age globally. Different tensions like accidents, conflicts, and occupational incidents lead to injury, affect public well-being, and cause economic and social concerns and disability. Almost 16% of the people who are injured become disabled for life (1-4). Orthopedic injuries are the most common type of injuries that may require further surgical interventions (5, 6). Nowadays there is sufficient information on the prevention of a major part of lethal or debilitating diseases; however, the resulting knowledge is not comprehensive enough to ensure effective disease and injury control (7). 

One of the main reasons for insufficient studies in this regard is the unavailability of the national data and statistics of injuries to trauma care givers, researchers, and institutes. Lack of the basic information is one of the main shortcomings in the execution of preventive plans in the field of injuries (8). The first step in controlling incidents is analyzing them to identify the underlying causes; therefore, development of a minimum data set (MDS) to collect data in a standard and integrated manner in a national level can be of a great importance (9). In line with the documented benefits of the MDS, some developed countries such as Denmark (NPRMDS 1987), Germany (MDIM 1995), Great Britain (NHS-MDS1993), the Netherlands (LIS-BDS1997), Australia (VEMD1995), Canada (MDIS1998), and New Zealand (NMDS-IS1992) have constructed their MDS on injuries (10); however, in Iran, as a developing country, there have not been any MDS for orthopedic injuries to the present times.

Information management collects and exchanges information among institutions and individuals using standard tools and a uniform language. Using such tools facilitates communication between individuals and institutions that are involved in patients care (11). Data collection is the most important part of information management and the MDS is a standard tool for collecting data (12, 13) that guarantees access to accurate and precise health data (14). With the use of the MDS, standard data, which are necessary for comparing and analyzing the activities to access new and credible information on the number of patients, diseases, new therapeutic methods, and their outcomes, are collected from all centers (15, 16). Many organizations use an MDS to develop documentation standards since it offers identical and uniform definitions and expressions for describing what has happened (17). Digitizing the data and their storage in databases has made the use of the MDS inevitable (18).

## 2. Objectives

The special nature of injuries and their outcomes necessitates standardized and nationwide defining and collecting injury-related data. The collected data satisfies the need of the individuals and institutions and provides necessary data for research on orthopedic injuries. The aim of this study was to develop a national MDS of the information management system for orthopedic injuries in Iran.

## 3. Materials and Methods

This descriptive and cross-sectional study was performed in 2013. The data were collected from hospitals affiliated with Tehran University of Medical Sciences, which had an orthopedic ward (Imam Khomeini, Sina, Shariati, Rasoul Akram, Firoozgar, and Shafa Yahyaian hospitals), medical documents centers (social security, medical services, armed forces, and assistance committee insurance institutions), four medical emergency centers, and legal medicine centers in the city of Tehran.

Data was collected from the records of patient with orthopedic injury in hospitals, legal medicine centers, medical documents centers, and the emergency form in medical emergency centers in 2012. In the hospitals, medical documents centers, and legal medicine centers, 10 samples of each injury according to S22-S99 entities of International Classification of Diseases 10th revision (ICD-10) were randomly selected and the data elements of the emergency forms in medical emergency centers were evaluated. A checklist was used to extract data elements.

In the next stage, a literature review was performed to retrieve relevant resources. Data sources for this stage were papers, reports, and forms on the internet and hard copies (texts, theses). In this stage, a checklist was used to extract the data elements.

To find materials relevant to the subject, search engines (Yahoo and Google), databases (Google Scholar, Cochrane, PubMed, and MagIran), and websites (Canadian Institute for Health Information (CIHI), Ontario Ministry of Health (OMH), and New Zealand Ministry of Health) were explored. Studies were identified by keywords including minimum data, Minimum data set form, orthopedic injury data, MDS, minimum data set, trauma registry form, and injury registry in Farsi and English languages. We mainly confined our search to materials published from 1990 to 2013. Sampling was not performed in this stage and all the relevant literature were retrieved and evaluated based on inclusion criteria and their data elements were entered into the checklist.

### 3.1. Inclusion and Exclusion Criteria

The searches were limited to literature in the English and Farsi languages. Papers, reports, and forms of research in the full text from valid sources and a clearly stated purpose published from 1990 to August 2013 as well as hard copies of available texts and theses were included. Non peer-reviewed papers, letter to editor, short communication, reports and forms retrieved from weblogs and abstracts with not accessible full text were excluded.

Review of the literature was performed until data saturation reached. A checklist was used to collect data that was assigned to two administrative and clinical data categories. Then the content of the final checklist was constructed by combining data elements extracted from reviewed patients’ records, the emergency forms in Iran, and data elements obtained from the literatures review. The data elements of the checklist were used to develop a questionnaire. Three columns of “No” and “Yes” (obligatory or optional) were added in front of each data element. At the end of each section, an empty box was provided to write the data elements that were necessary to register according to experts’ opinion.

The content validity of the questionnaire was evaluated using the comments from experts in the field of health information management, orthopedic surgery, general practitioner, insurance, legal medicine, and emergency medicine (a total of 12 persons, consisting of two experts in each field). To ensure the reliability of the questionnaire, it was completed by ten of the aforementioned experts; they were requested to complete the questionnaire for the second time after one week. The collected data were analyzed with SPSS 16 (SPSS Inc., Chicago, IL, USA). Spearman's rank correlation coefficient was used to evaluate the reliability of the questionnaire, which showed a coefficient of 85%. 

To determine the MDS of the information management system for orthopedic injuries, the final data elements were chosen by 30 samples of attended experts (demographic characteristics of the samples are described in Table 1) through decision Delphi technique in two rounds. Deciding on included data elements were based on the agreement level. In this way, data elements with less than 50% agreement were excluded in the first round and those with more than 75% agreement (both obligatory and optional “Yes”) were included in the primary round. Those with 50% to 75% agreement were surveyed in the second round and if there was 75% consensus over a subject, it was regarded as a final data element.

**Table 1.** Demographic Characteristics of the Samples

Samples	Frequency
**Information Management Experts (n = 6)**	
**Sex**	
Male	4
Female	2
**Age group, y**	
20-30	1
30-40	3
40-50	2
50 <	0
**Education**	
PhD	6
**Academic field**	
Health Information management	6
**Work experience, y**	
< 6	1
6-10	3
11-15	0
15-20	2
20 <	0
**Orthopedic Surgeons (n = 5)**	
**Sex**	
Male	5
Female	0
**Age group, y**	
20-30	0
30-40	2
40-50	2
50 <	1
**Education**	
*Specialist*	5
**Academic field**	
Orthopedist	5
**Work experience, y**	
< 6	2
6-10	2
11-15	0
15-20	0
20 <	1
**Legal Medicine Specialists (n = 4)**	
**Sex**	
Male	4
Female	0
**Age group, y**	
20-30	0
30-40	1
40-50	2
50 <	1
**Education**	
*Specialist*	4
**Academic field**	
*legal medicine*	4
**Work experience, y**	
< 6	1
6-10	2
11-15	0
15-20	1
20 <	0
**General Practitioners (n = 5)**	
**Sex**	
Male	3
Female	2
**Age group, y**	
20-30	0
30-40	3
40-50	2
50 <	0
**Education**	
General physician	5
**Academic field**	
General physician	5
**Work experience, y**	
< 6	0
6-10	3
11-15	2
15-20	0
20 <	0
**Insurance Experts (n = 5)**	
**Sex**	
Male	5
Female	0
**Age** **group, y**	
20-30	0
30-40	2
40-50	2
50 <	1
**Education**	
General physician	2
Master of sciences	3
**Academic field**	
General physician	2
Nursing	3
**Work experience, y**	
< 6	0
6-10	2
11-15	2
15-20	1
20 <	0
**Emergency Medicine Specialists (n = 5)**	
**Sex**	
Male	5
Female	0
**Age group, y**	
20-30	2
30-40	3
40-50	0
50 <	0
**Education**	
**Specialist in Emergency Medicine**	5
**Academic field**	
Emergency Medicine	5
**Work experience, y**	
< 6	5
6-10	0
11-15	0
15-20	0
20 <	0

## 4. Results

The MDS of the orthopedic injuries was assigned to two categories; Administrative data with six and clinical data with 17 classes. Total numbers of data elements collected for administrative and clinical categories were 203 and 375, respectively. After applying the two stages of the decision Delphi technique, the final set of data elements was determined for administrative category with 142 and clinical category with 250 (Tables 2 and 3).

The administrative data classes were as follows: demographic data of the patients including first and last name, age, sex, and living status; providers’ identification data including the data of the care provider institutions, individuals, or experts; insurance data including information essential to reimburse the costs of hospitalization and treatment by insurance companies; legal data including data elements that had legal specifications and were essential to insurance claims, indemnity, disability, and legal claims; cause data including the data elements regarding the cause of the orthopedic injury (the intent, the name of the causing agent, and the type of activity); and place data including data elements concerning the location of the accident some of which were the type of the place (educational, industrial, public, etc.), its ownership type (private, governmental, etc.), and its geographical specifications (Table 4).

The clinical data classes included diagnostic data with two subclasses of general data for diagnosis and data elements for injuries based on S22-S99 entities of ICD-10. Data elements were regarded as a whole in the first subclass. In the second subclass, for each injury, diagnostic data elements were identified according to S22-S99 entities in detail, which included fracture of rib(s), sternum, thoracic spine, lumbar spine and pelvis, shoulder and arm fracture, elbow, forearm, wrist, and hand fracture, hip and femur fracture, and lower leg injuries. The data of this subclass were extracted from patients’ records, data elements of conducted studies, and injury-related entities in chapter 19 of ICD-10.

Emergency data were related to the medical emergency centers and emergency departments of the hospitals. The emergency form and the emergency department records of the patients were major resources of data in this class. Anesthesia data showed data about the type, status, duration, and drugs used for anesthesia. Procedure data included surgical and nonsurgical procedures. Medical history includes patient, family, drug, and diet history of the patient. Consultation data included data elements related to the patients’ physicians and consultants. Orders were data elements of the physicians’ order(s) for the patients.

X-ray data included invasive and noninvasive radiological procedures. Lab tests data included the data elements regarding the laboratory tests and pathologic examinations. Drugs data included the prescription, dosage, amount, and duration of the drugs administration. Instrument data included the devices used for fixation or orthopedic surgery. Blood products data included data elements on the type, unit, number, and serial number of each blood packs. Nursing notes included data elements of the nurse notes, interventions, observations, controlling, confirming physicians’ orders, and patients’ education. Discharge condition included data elements about the outcome of care, patient status on discharge, medical and follow-up orders, place of follow-up visits, and date of the next follow-up.

Follow-up included follow-up request for completing treatment, type of rehabilitation, and performed procedures. Death data included data elements on death cause as well as primary, underlying, and external causes of death according to ICD-10, autopsy, and organ donation. Transfer data included data elements on patient transfer to another department in the hospital, to another hospital, or to another city (Table 5).

**Table 2.** Clinical Data Classes for a Minimum Data Set for Orthopedic Injuries

Data Classes	The Number of Data Elements	First Round of Delphi			Second Round of Delphi			Final
		< 50%	50-75%	75% <	< 50%	50-75%	75% <	
**Diagnostic**	56	14	11	31	5	0	6	37
**Emergency**	78	19	16	43	9	0	7	50
**Anesthesia**	19	3	4	12	3	0	1	13
**Procedure**	71	17	15	39	9	0	6	45
**History**	11	3	2	6	2	0	0	6
**Consultation**	9	2	1	6	1	0	0	6
**Order**	15	3	1	11	1	0	0	11
**X-ray**	22	5	2	15	1	0	1	16
**Lab test**	9	3	1	5	0	0	1	6
**Medication**	8	1	2	5	1	0	1	6
**instrument**	11	2	3	6	2	0	1	7
**Blood product**	8	2	1	5	1	0	0	5
**Nursing**	13	1	3	9	2	0	1	10
**Condition of discharge**	9	0	3	6	1	0	2	8
**Follow up**	9	2	1	6	1	0	0	6
**Death**	11	2	2	7	1	0	1	8
**transfer**	16	3	4	9	3	0	1	10
**Total**	375	82	72	221	43	0	29	250

**Table 3.** Administrative Data Classes for a Minimum Data Set for Orthopedic Injuries

Data classes	The Number of Data Elements	First Round of Delphi			Second Round of Delphi			Final
		< 50%	50-75%	75%<	< 50%	50-75%	75% <	
**Demographic**	60	12	9	39	4	0	5	44
**Provider ID**	38	7	8	23	5	0	3	26
**Insurance**	45	9	8	28	6	0	2	30
**Legal**	42	10	6	26	4	0	2	28
**Cause**	11	2	2	7	1	0	1	8
**Place**	7	0	2	5	1	0	1	6
**Total**	203	40	35	128	21	0	14	142

**Table 4.** Examples of Administrative Data Elements for a Minimum Data Set for Orthopedic Injuries

Data Class
Demographic data
Patient’s name
Patient’s family
Father’s name
Marital status
Medical record number
Sex
Age
Birth date
**Provider (Organizational, Personal) identification data**
Facility Name
Facility/hospital address
Organizational dependency
Patient’s number
Specialty
Provider address
**Place data**
Injury Place
Place address
Place possession
**Insurance data**
Method of payment
Payment program identifiers
Charges, payments
Responsibility For Payment
Serial number
Insurance type
**Legal data**
Advance directives
Allergy records
Consent forms for care, treatment, and research
Organ donation
**Cause data**
Cause of orthopedic injury
Intent
Date of incident
The name of incident cause

**Table 5.** Examples of Clinical Data Elements for a Minimum Data Set for Orthopedic Injuries

Data Class
**Diagnostic data**
Chief complaint
Primary diagnosis
Final diagnosis
Other diagnosis
Body part injured
Fracture direction
**Emergency data**
Injury Date
Injury time
Transported by land ambulance
Transported by air ambulance
Dispatch date
Dispatch time
**Anesthesia data**
Kind of anesthesia
Anesthesia drug
Start of anesthesia
End of anesthesia
anesthesia time
Anesthesia risk level
**Procedure data**
Date of procedure
Procedure name by ICD-9-CM
Date of surgery
Start of operation
End of operation
Internal fixation
External fixation
**History**
Past disease history
Drug or food allergy
Family history
Fracture history
**Consultations**
Consultation with service requests
Type of consultation
Consultation date
Consultation time
**Orders**
Inpatient order
Blood reserve order
Radiography orders
Dietary orders
Order date
Order time
**Follow up**
Request Rehabilitation
Type of Rehabilitation
Number of meetings
activity
**Death data**
The main cause of death
Underlying cause of death
External causes of death
Date of death
Place of Death
**X-rays**
Type of requested radiography
Limb’s name
Limb’s direction
Date of radiography
Radiologist diagnosis
**Lab-tests**
Requested test
Pathology reports
Tests Results
Pathology results
Date
**Medications**
Name and type of medications
Value
Dose
Type of Prescription
**Instrument**
Name of the requested
instrumentsSize
Serial number
date of produced
Manufacturer
**Blood products**
Blood request
Blood type
Unit
Blood pack serial number
**Nursing note**
Nursing report
Date of report
Time of report
Confirm orders by nurses
Patient education
**Conditions of discharges**
Discharge date
Discharge time
Medication instructions at
discharge
Places to visit follow up
**Transfer data**
Cause of dispatch or transfer
Transfer date
Origin hospital
Transfer time
Destination hospital

## 5. Discussion

Iran is among the countries with the highest rate of accidents and occupational injuries. Many people were also injured in the Iran-Iraq War. Therefore, the treatment of victims of war and current events has made Iran amongst the top countries in the science of the orthopedics. Primarily results of this study showed that the orthopedic data were not collected in a standard way and developing an MDS was required for orthopedic injuries.

Organized and nonorganized data are accessible in every organizational structure and therefore, the need for managing the data is evident (19). Injuries are a very common type of noncommunicable diseases in our century that occur due to expansions and developments in living environments and conditions. As a result, legal outcomes and claims that may ensue require precise and accurate data collection and registry (1, 8). In this research, the MDS for orthopedic injuries was assigned to clinical and administrative data based on the previously conducted studies, standards set by American National Standard Institute (ANSI), and reference books (11, 20-23).

No exclusive and standard MDS has been developed to register orthopedic injuries data in Iran. It is evident that standardization leads to conceptual interoperability (11). Therefore, standard definitions should be used for data with minimal free text (17).

Neglecting the special items of insurance resulted in incomplete registry of the data required by medical documents centers. Incomplete registration of the documents by treatment teams were one of the major reasons for deduction (24, 25). Laing stated that the MDS prepared a framework for developing the necessary conditions for comprehensive documentation of the records (18). Cai et al. stated that the accuracy of the MDS in identifying hospitalizations and payment source varied across the study states, which should be evaluated carefully with regard to the intended uses of the data (26).

Data elements for care providers in Iran were not exhaustive. Data elements for registering the specifications of care centers help to their better identification and facilitate patient-care center relationship. Ahmadi et al. concluded that there were no standards as what nursing items to register in the electronic health records of the patients in Iran (23). Registering forensic data elements are important in injuries since they mostly result in legal claims and legal authorities require accurate data. 

About 75% of deaths occur at the accident scene and during transferring the patient to the hospital (27). Hence, development of an MSD to improve the organization of prehospital emergency for providing emergency medical services plays an important role in timely and proper response to incidents (28). The results of a study performed by Lai showed that the AIDS MDS improved health through data exchange and was capable of changing the traditional interactions of care givers (29).

Registering the data elements of anesthesia and the performed procedures results in better follow-up of the patient care and provides the necessary data for insurance companies (25). Primary care specialists believed that the use of the MDS, electronic drug prescription, and electronic drug management enhanced the continuity of care (30). The results of a study by Karimi et al. showed that different forms should be designed for accurate data registry in organ donation to document all data and evaluations (22). Registering data elements of the medical and family history of the patients, radiologic data, lab tests, physician’s orders, prescribed medicines, required blood products, and devices used for fixation improves patient care and lowers treatment costs.

If data elements are documented when the patient is transferred to another ward, hospital, or city for any reason, it would help to make better decisions regarding patient transfer, equipment of the health centers, and management of available resources. Registering the data elements of primary and underlying cause of death results in identification of the causes of death and lowering injury-related mortality.

To identify the main causes and control injury, data should be collected in a standard manner at a nationwide level. Research has shown that some essential data elements, which are required by different institutions or care givers and are registered in other countries, are not collected in Iran. Comprehensive information on the cause and mechanism of injuries enables public health authorities to inform the public about the injuries and prevent their occurrence. In this way, every injury presents an opportunity for preventing a similar one.
